# The Narrative Review: Advancements in Heart Failure Diagnosis and Management using Artificial Intelligence: A New Era of Patient Care

**DOI:** 10.2174/011573403X369978250818060357

**Published:** 2025-09-11

**Authors:** Sunchandandeep Singh Brar, Meenakshi Reddy Yathindra, Juan Sebastian Arias Arango, Erika Aguirre Gutierrez, Fawaz Aldoohan, Princejeet Singh Chahal, Apo Youssef, Mahima Srinidhi Narra, Mahesh Babu Tatineni, Jorge Manuel Aldea Saldaña, Gabriel Ramírez Torres, Pallavi Shekhawat, Vyapti Dave

**Affiliations:** 1 Department of Internal Medicine, China Medical University, Shenyang, China;; 2 Department of Internal Medicine, Kasturba Medical College, Mangalore, India;; 3 Department of Internal Medicine, Central University of Valle del Cauca, Tuluá. Colombia;; 4 Department of Internal Medicine, Universidad Autónoma De Guadalajara School of Medicine, Guadalajara, Mexico;; 5 Department of Internal Medicine, Kuwait University, American Academy of Research and Academics, Kuwait City, Kuwait;; 6 Department of Internal Medicine, Adesh Institute of Medical Sciences and Research, Bathinda, Punjab, India;; 7 Department of Internal Medicine, Faculty of Medicine & Medical Sciences, University of Balamand, Tripoli, Lebanon;; 8 Department of Internal Medicine, Dr. PSI Medical College, Chinna Avutapalli, Vijayawada, Andhra Pradesh, India;; 9 Department of Internal Medicine, Faculty of Health Sciences, Peruvian University of Applied Sciences, Lima, Peru;; 10 Department of Internal Medicine,Tecnológico de Monterrey Medical School, Monterrey, México;; 11 Department of Obstetrics & Gynecology, Employees’ State Insurance Post Graduate Institute of Medical Sciences, Delhi, India;; 12 Department of Internal Medicine, Gujarat Medical Education and Research Society (GMERS) Valsad, Gujarat, India

**Keywords:** Heart failure, artificial intelligence, machine learning, deep learning, healthcare, electrocardiogram, AI algorithms

## Abstract

Heart Failure (HF) is a prevalent medical illness worldwide that affects millions and is a substantial economic burden. Its epidemiological impact is on the rise due to factors such as the ageing of the population, increasing rates of diabetes and hypertension, and better survival post-myocardial infarction. Some limitations in HF management include diagnostic challenges, sudden progression of the disease, and rising rates of readmission. Continuous monitoring and limited therapeutic interventions add further complexity to care. Artificial Intelligence(AI) is essential in health care and has provided solutions for improving HF management. Techniques like machine learning and deep learning enhance clinical decision-making and patient care. AI helps physicians diagnose HF more precisely through the analysis of imaging and electrocardiograms. Additionally, the patients' risk is calculated using various AI algorithms to develop personalized treatments for each individual. AI will help healthcare providers identify problems early and select appropriate therapies, leading to better outcomes. Further areas for improvement include enhanced data integration, predictive accuracy, patient engagement, data privacy and ethics, as well as integration with clinical workflows. AI technologies will continue to evolve in managing and treating HF; ongoing exploration and development are crucial for its optimization. This review outlines the current progress and potential of AI in the future to ensure better patient care and healthcare practices.

## 1 INTRODUCTION

Heart failure (HF) is a complicated clinical illness caused by defects in the structure and/or function of the heart, which prevent the heart from pumping enough blood through the circulatory system to meet the body's needs. Shortness of breath, exhaustion, edema (arm and leg swelling), chest crackles, or elevated jugular vein pressure are some symptoms that may manifest [1, 2]. With an estimated incidence rate of 1.3%, it is a significant contributor to morbidity and healthcare expenditures globally, posing a challenge to health systems worldwide to manage these large numbers [3]. Furthermore, because the symptoms of HF are mainly non-specific, diagnosing the ailment can be difficult (2016), which makes it a dangerous medical condition [4]. Fortunately, new Artificial Intelligence (AI) developments have emerged in response to these trends, potentially altering how we tackle HF [5-11].

A field of computer systems that can function with varying degrees of automation to analyze vast amounts of data, provide outputs like content, forecasts, recommendations, or judgments, and solve issues that often call for human intelligence is known as artificial intelligence (AI) [12, 13]. AI has advanced significantly, particularly in the last several years, due to advancements in its subset of “Machine Learning” (ML), which has produced more effective “Deep Learning” (DL) [14, 15].

**Fig. (1):**
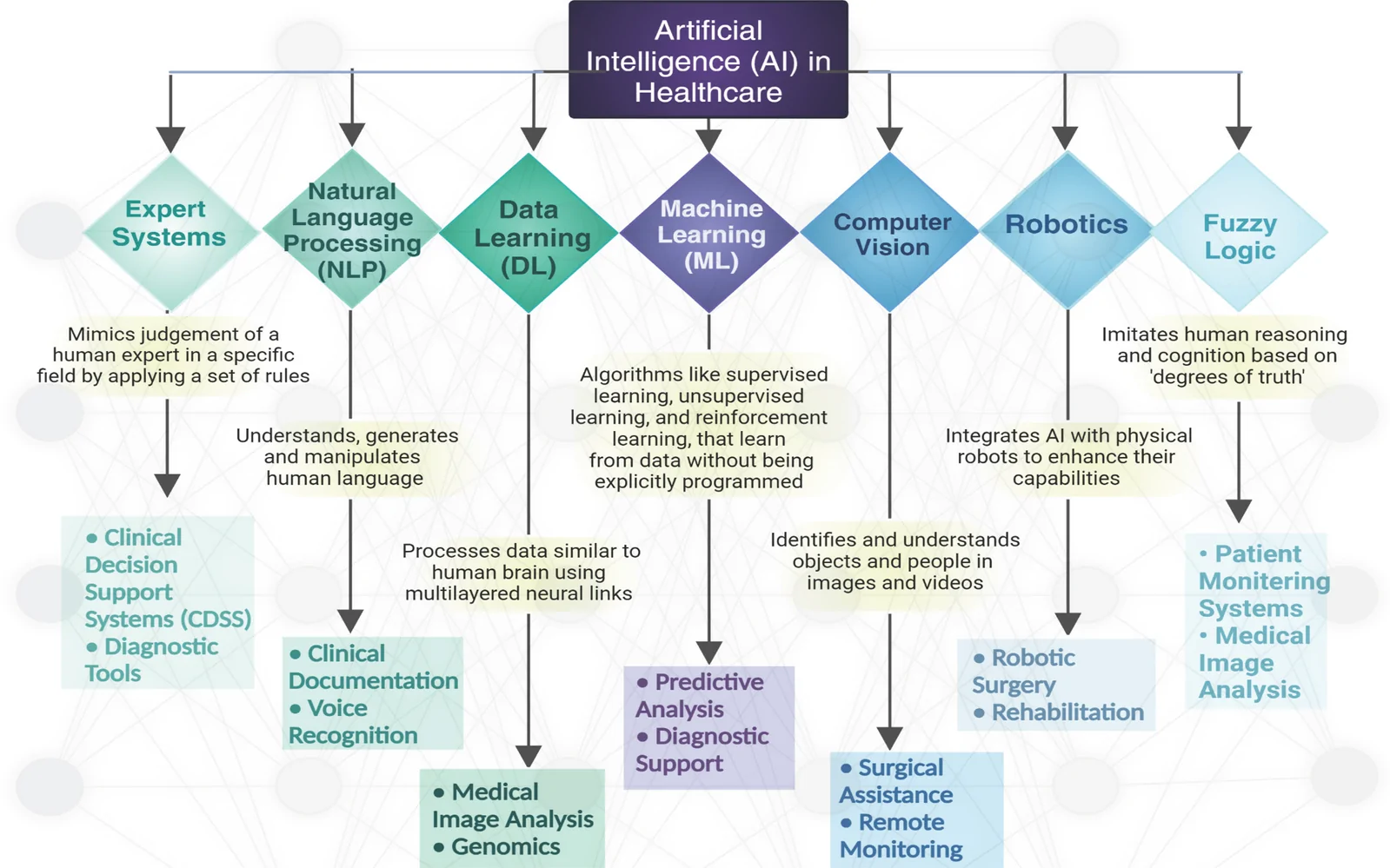
AI role in healthcare.

AI developments have the potential to completely change how doctors diagnose, treat, and manage heart failure [16, 17]. AI-enhanced ECGs, for example, have demonstrated high diagnostic accuracy in identifying left ventricular systolic dysfunction (LVSD). This condition frequently results in heart failure with a reduced ejection fraction (HFrEF) [18, 19]. AI-based techniques have now gained widespread use in cardiac imaging, a crucial aspect of assessing heart failure and facilitating quicker image capture, analysis, and clinical decision-making. Additionally, real-time monitoring of a patient's vital signs and symptoms can be made possible by AI-powered systems, alerting the patient and the healthcare provider to any concerning changes (Fig. **1**) [20, 21].

### 1.1 Methodology

In this review, an extensive literature search was conducted in numerous databases, including PubMed, Scopus, Medline, and Google Scholar, to find relevant studies on diagnosing and treating heart failure using Artificial Intelligence (AI). The search strategy involved combining Medical Subject Headings (MeSH) and free-text terms about “heart failure,” “artificial intelligence,” “machine learning,” and “deep learning.”We applied the inclusion and exclusion criteria for this review.

Studies were considered based on their relevance toward AI applications for diagnosing or treating heart failure, published between 2017 and 2024, and authored in English. We excluded studies that did not apply AI, non-English papers, and studies that lacked data richness or had poor methodological quality.

## 2 IMPLICATION OF AI IN THE DIAGNOSIS OF HEART FAILURE

Artificial intelligence closely resembles the human brain, and its algorithms can assist physicians in their day-to-day practices; its applications include predicting, pre-diagnosing, correcting errors like medication errors, and preventing misdiagnosis [22, 23]. AI uses algorithms to continuously learn new data by connecting with preexisting data or manually encoding new data [24]. AI has greater predictive power in identifying risk factors for the primary prevention of heart diseases than the American Heart Association (AHA) and American College of Cardiology (ACC) institutions, as the machine learning algorithms can predict 7.6% more incidents and consider additional 22 risk factors. Another study found the application of the Neural Network Model (NNM) on the clinical data of 40 persons, and it predicted the possibility of cardiac failure with a precision rate of 85%. Doctors are better equipped by using AI in MRIs, ECGs, Echocardiography, angiography, and other imaging and diagnostic modalities to diagnose rare and complicated cardiovascular diseases with better accuracy and act as a valuable tool, particularly in Heart Failure(HF) patients [25, 26]. Garan AR *et al*. performed Real World Evidence (RWE) search when they applied AI to Electronic Health Record data obtained from 2015 to 2019. Of 1155 patients, 472 patients had HF, and when applied to raw unstructured data obtained from health records through advanced approach, the highest level of accuracy was obtained in identifying HF with varying ejection fraction in patients (recall percentage =96%, precision =94.7%). Even Left Ventricular Ejection Fraction (LVEF) values were obtained compared to when they applied AI to structured diagnostic codes and problem lists with standard inquiry questions. The authors pointed out the significance of accurate phenotyping of data to achieve accuracy in test results and the need for AI to RWE [27]. Researchers implemented AI using the” 2013/2016 Guidelines for the Management of Heart Failure,” which used answer-set programming and declarative programming to mimic human reasoning. As demonstrated in Fig. (**2**), this pilot study of the heart failure advisor system was able to provide recommendations and compare them to the attending cardiologist’s recommendations with 90% accuracy. The rest of the 10% attributed to a lack of information inputs for the system, differences in practice styles, and the need for additional programming [28].

### 2.1 Use of AI in X-rays

Celik A. *et al*. applied AI to interpret thoracic X-rays of 10,100 patients to diagnose heart failure; depending on the results, each group went on to get further diagnostic tests. Dyspnea is one of the chief clinical symptoms in most patients with HF, and X-rays are a widely used, standard, primary screening modality available worldwide, even in low-resource, remote areas. Some signs programmed by the AI algorithm to detect in X-rays were cardiothoracic ratio>50%, lung congestion, bilateral pleural effusions, hilar haziness, Kerley B lines, *etc*. According to the 2021 European Society of Cardiology(ESC) guidelines, an 84% precision rate was found in more than half of the patients diagnosed with HF with preserved ejection fraction (diastolic HF). ART-IN-HF trial shows the use of AI to triage HF patients. It successfully detected 54.9% of the undiagnosed population with Heart Failure with preserved Ejection Fraction (HFpEF), making it a useful rapid screening tool [29].

Matsumoto (*et al*.) employed deep learning algorithms in chest X-rays to narrow the gap between diagnosing heart and pulmonary conditions using deep learning. The study will utilize deep learning technology to diagnose cardiac failure based on X-ray images and improve the accuracy of their interpretation. Nine hundred fifty-two chest x-rays and heatmap images were sourced from the National Institutes of Health(NIH) databases and analyzed by using learning algorithm. Of those, 260 were identified as normal, 378 as 'heart failure,' and the remaining X ray images were removed due to incorrect identification. The overall accuracy was 82%, with a sensitivity and a specificity of 75% and 94.4%, respectively [30].

### 2.2 Wireless Cardiac Pressure and Impedance Monitoring Sensors

V-LAP is a battery-less cardiac monitoring device that measures left atrial pressure; increased values are one of the early and specific signs of Heart failure [31]. In 2019, an article found that Mobile cardiac telemetry (MCT) devices have the highest diagnostic precision of 61% in identifying abnormal heart rhythms over long periods compared to 23% in event monitors and 24% in Holter monitors [25, 32]. This method calculates intrathoracic fluid levels using the thoracic impedance marker, which is calculated by surface electrodes placed on the patient. It is more sensitive than measuring weight change to indicate decompensation. However, further research is needed to improve the sensitivity of this technology [25, 33, 34].

The St. Jude Medical Team developed a pulmonary artery pressure monitoring implant called CardioMEMS. This implant measures left ventricular pressure using an intra-atrial sensor placed in the patient's heart, thereby preventing potential complications from decompensation by alerting specialists. Future research could potentially focus on developing a more promising implant that monitors venous blood oxygenation and right ventricular pressure, targeting two vital parameters: patients' left atrial pressure and minute ventilation [35].

### 2.3 Diagnosing Heart Failure using ECGs Enhanced by AI

The interpretation of ECGs depends on the clinician's level of experience and proficiency; however, analyzing each ECG for thousands of possible diseases, depending on the alterations, is not feasible in everyday practice. With its graphic analysis capabilities, learning power, and complex algorithms, AI can detect even slight changes in ECG and compare them with thousands of pre-existing datasets to develop a pre-diagnosis for all patients; hence, the early detection of various cardiovascular disorders is made possible [25, 36]. One of the initial prospective studies to study the use of Deep Learning algorithms to screen patients with low ejection fraction in HF was the EAGLE trial (ECG- utilizing AI algorithm-guided screening to detect Low Ejection Fraction) [35, 37].

AI's deeply trained neural network can even detect subtle changes in atrial fibrillation within a normal sinus rhythm. Attia *et al*. developed an AI ECG model with an accuracy of 88.3% [38]. AI ECG can also detect and predict future risks of Asymptomatic Left Ventricular Dysfunction in patients without even undergoing an Echocardiogram, hence warranting early diagnosis and treatment using a cost-effective method [39, 40]. Yao (*et al*.), created an artificial model to detect CHF with reduced Ejection fraction <50% from ECGs, thereby improving diagnosis by 32% in primary care settings [37]. Vaid *et al*. formatted an AI algorithm of the deep learning model (DLM) of ECG to detect changes in bilateral ventricular function; the model was able to predict right ventricular systolic dysfunction accurately and detected patients presenting with <=40% left-sided ejection fraction, hence a valuable screening tool in remote and low-cost settings [24, 41]. Kwon (*et al*.), developed a valuable deep learning technology that utilizes ECG to identify, screen, and predict risk in patients with Heart failure presenting with preserved ejection fraction (HFpEF) by focusing on R waves and T waves in ECGs [42]. People identified by DLM have increased morbidity of HFpEF in the upcoming 24 months (Relative Risk (RR) =33.6, *p*<0.001), hence a reliable tool in screening patients [42]. The interpretation of ECGs for arrhythmias and CHF can be facilitated by AI models, including Support Vector Machines, Random Forest models, Artificial Neural Networks (ANN), and the C4.5 decision tree model. Hussain L. found SVM to have the best prediction and accuracy scores compared with other algorithms employed to detect CHF [25].

In 2015, Zheng *et al*. found that Artificial Neural Networks and Hidden Markov Models were superior to Least Squares Support Vector Machines [25, 43].

A deep learning AI algorithm, when incorporated into -lead ECG-enabled stethoscopes during routine clinical examinations, particularly over the pulmonary valve during auscultation (sensitivity: 84.8%) and handheld positions, was able to detect patients with low ejection fractions (<40%), hence providing a cost-effective point-of-care screening model [44, 45]. The model yielded false positives in patients with higher ejection fractions (41-50%). This model has a high negative predictive value; utilizing a logistic regression model or biomarker analysis can upgrade the technology for better prediction accuracy [46]. HF is one of the most dreaded and frequently developed devastating complications seen in patients with Chagas' disease; machine learning AI-ECG technology was used to screen for left ventricular systolic dysfunction in early HF, and results show 83% accuracy of prediction with a high negative predictive value of 97%. Significant Q wave abnormalities and ST-T changes can increase morbidity. The application of AI in diagnosing cardiac failure in suspected cases with multiple comorbidities, particularly in remote or inaccessible regions, is both cost-effective and reliable. Results may be augmented with NT-proBNP biomarker testing [47].

### 2.4 Heart Failure With Preserved Ejection Fraction (HFpEF)

AI is a valuable tool for addressing heart failure with preserved ejection fraction (HFpEF). Rapid pre-screening in patients with suspected disease based on intra-beat dynamics of echocardiograms allows for rapid and accurate assessment of HFpEF [48]. Echocardiogram-based learning models presented through artificial intelligence (EchoGo Heart Failure v2) outperformed H_2_FPEF and HFA-PEFF scores, suggesting that integrating AI into clinical diagnosis could improve the identification of HFpEF [49].

Fig. (**3**) illustrates an example of how machine learning can perpetuate or even exacerbate diagnostic uncertainties. The two currently used algorithms for HFpEF diagnosis are HFA-PEFF and HF_2_FPEF. Thus, the HFpEF clusters obtained using unsupervised machine learning will strongly depend on the algorithm used, and the diagnostic uncertainty will ultimately lead to cluster definition uncertainty. These two algorithms disagree in approximately 40% of the cases [35].

Natural Language Processing (NLP): The application of NLP in electronic health records has demonstrated potential to enhance the detection of HFpEF. A study showed that many patients who met the European Society of Cardiology diagnostic criteria for HFpEF had not been formally diagnosed, indicating that NLP techniques can identify patients who may benefit from expert clinical review [50].

In the past, Chiou *et al*. trained a DL model for diagnosing HFpEF on a smaller dataset of 4-chamber echocardiography videos, achieving greater accuracy, sensitivity, and specificity [48, 51]. Akerman *et al*. used a sizable and comprehensive dataset that covered multiple centers and nations, meticulously developed and tested their algorithm in pertinent subgroups, and performed validation on independent and separate holdout datasets [49]. Additionally, the authors developed heatmaps to identify the most important echocardiographic features that the algorithm used to distinguish between patients with HFpEF and those with non-HFpEF, thereby helping to “open the black box” of DL algorithms. Several unanswered questions and potential research topics remain. The studies mentioned above still use suboptimal methods to confirm a definitive HFpEF diagnosis [51].

### 2.5 Integrating AI into Imaging Investigations

**Fig. (4):**
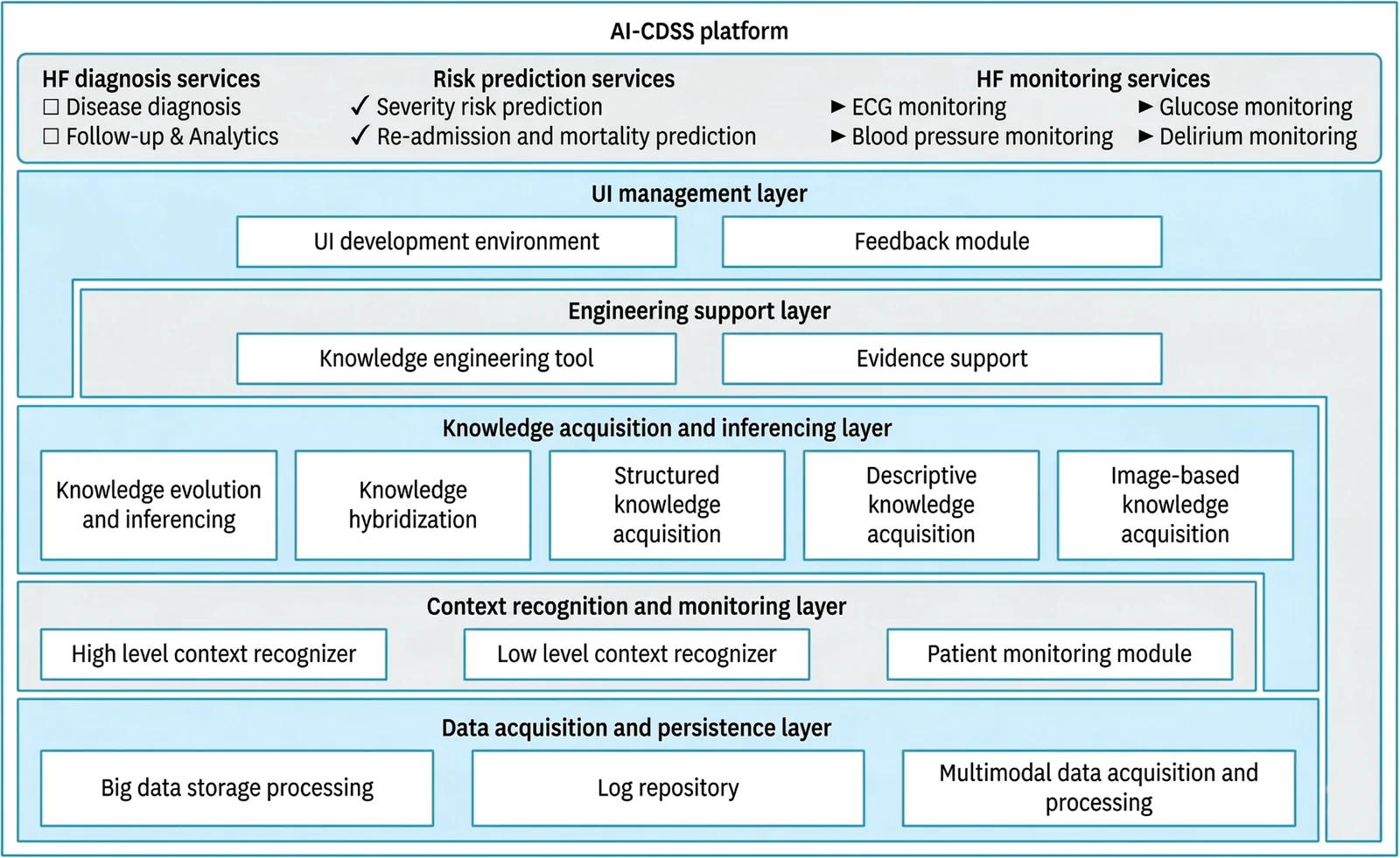
Hybrid AI-CDSS (expert system and machine learning) for diagnosis of heart failure. Reproduced from ref [57]. Under the terms of the Creative Commons Attribution Non-Commercial License (https://creativecommons.org/licenses/by-nc/4.0) Copyright © 2024. Korean Society of Heart Failure. **Abbreviations:** AI-CDSS = artificial intelligence-clinical decision support system; HF = heart failure; ECG = electrocardiography; UI = user interface.

Based on Late gadolinium enhancement image analysis, Deep Learning Convolutional Neural Networks can also distinguish and quantify normal myocardial tissue from infarcted tissue, especially in the left ventricle. AI and its application in nuclear cardiovascular diagnostics for HF patients are yet to be researched and developed [35, 52]. Currie (*et al*.), through artificial neural network interpretation, found that 123-iodine meta-iodobenzylguanidine single photon emission computed tomography imaging with a planar washout of >30% from non-infarcted myocardium and a decline of >10% of left-sided ejection fraction is found to be the best predictor of cardiac events in heart failure patients [53]. Supervised and unsupervised DL, ML, and ANN technologies are used in cardiac imaging techniques to identify and assess cardiovascular diseases. Machine learning algorithms are used in Cardiac MRI for ventricular segmentation to yield imaging biomarkers to predict CHF. AI in 3D imaging and reconstruction can be a valuable tool for physicians to visualize the heart chambers, primarily the right ventricular chamber. Medvedofsky *et al*. also used it to calculate left ventricular end-diastolic volume, ejection fractions, and left atrial volume at the end of ventricular systole using an automatically adaptive artificial analytical model [54]. A heart monitor is an AI software that assists physicians in interpreting echocardiograms by analyzing parameters like heart chamber volumes and ejection fractions. It is widely used compared to manual interpretation. However, Luo X *et al*. found that the automated device slightly overestimated the heart chamber volumes in the study conducted on a Chinese population. In contrast, the rest of the parameters were the same [55]. Convolutional neural networks (CNNs) are used to analyze and interpret 2D and 3D images, increasing the simplicity of the extraction of relevant information from them. Generative adversarial networks can convert cardiac magnetic resonance images into computed tomography images to detect abnormalities in vessels and retrieve image products from the data [56]. For the early identification of HF in at-risk patients, physicians could benefit from AI algorithms and develop an AI-based clinical decision support system (AI-CDSS) (Fig. **4**) [57]. It displayed a phenomenal diagnostic accuracy of 98% for heart failure, surpassing that of non-heart failure specialists (76%). This indicated that the AI-CDSS may be helpful particularly for HF diagnosis, mainly when access to HF specialists is limited. Intelligence-Clinical Decision Support System (AI-CDSS) is an amalgamation of Machine Learning and expert-driven knowledge models created to diagnose HF in remote and primary care settings where specialists are unavailable. In Expert driven knowledge acquisition, physician’s decisions were incorporated into the Decision tree. In Machine learning-driven rule generation, five different algorithms were used: J48, random forest, decision tree, Chi-squared automatic interaction detection (CHAID), and classification and regression tree (CART). This web-based application can be used in everyday practice with great feasibility due to its accuracy in diagnosing various types of heart failure, with the net accuracy of the hybrid model being 98.3% [25, 58]. A machine learning model was formulated to accurately quantify and automate LVEF similar to the values that were manually calculated by clinicians, hence saving valuable time and efforts; even nurses were able to dynamically monitor LVEF values without a professional radiologist and alert physicians to any changes, which led to better patient outcomes [24, 56].

### 2.6 AI in Wearable Electronic Devices

Wearable health monitoring technology and its sensors have been incorporated into smartphone apps with which patients and physicians can monitor vitals, levels of physical activity, and other data; *e.g.*, abrupt early morning weight gain can be a potential sign of early decompensation in HF patients. Bluetooth connectivity to weighing scales automatically uploads data into patients' electronic health records, thereby alerting physicians early about any complications. The increasing affordability of these external monitoring devices has made them quite popular in the remote monitoring of HF patients. AI-powered Atrial fibrillation (AF) detection watches, like versions 4 and 5 AppleWatch, sensors like Kardia, AliveCor, and now six-lead ECG like KardiaMobile 6L have been developed to detect any arrhythmias in HF patients. An early dose of anticoagulants can prevent the risk of stroke in these patients. AI-trained algorithms can quantify potassium levels in patients taking diuretics based on Deep Learning data from tall T waves in patients with hyperkalemia, hence the name “bloodless blood tests” [33]. The Internet of Things (IoT) is an electronic software that combined with A, allows physicians to monitor patients' vitals remotely and warn about impending Heart attacks. Cardiac Resynchronization Therapy (CRT) technology ascertains the level of intra-thoracic impedance. The The DL-based early warning system (DEWS) demonstrated better accuracy in a hospital setting for identifying patients at risk of cardiac arrest, with 75% sensitivity, compared to logistic regression and random forest models. It is preferable over track and trigger systems and rapid response systems due to its lower rates of false alarms.

**Fig. (5):**
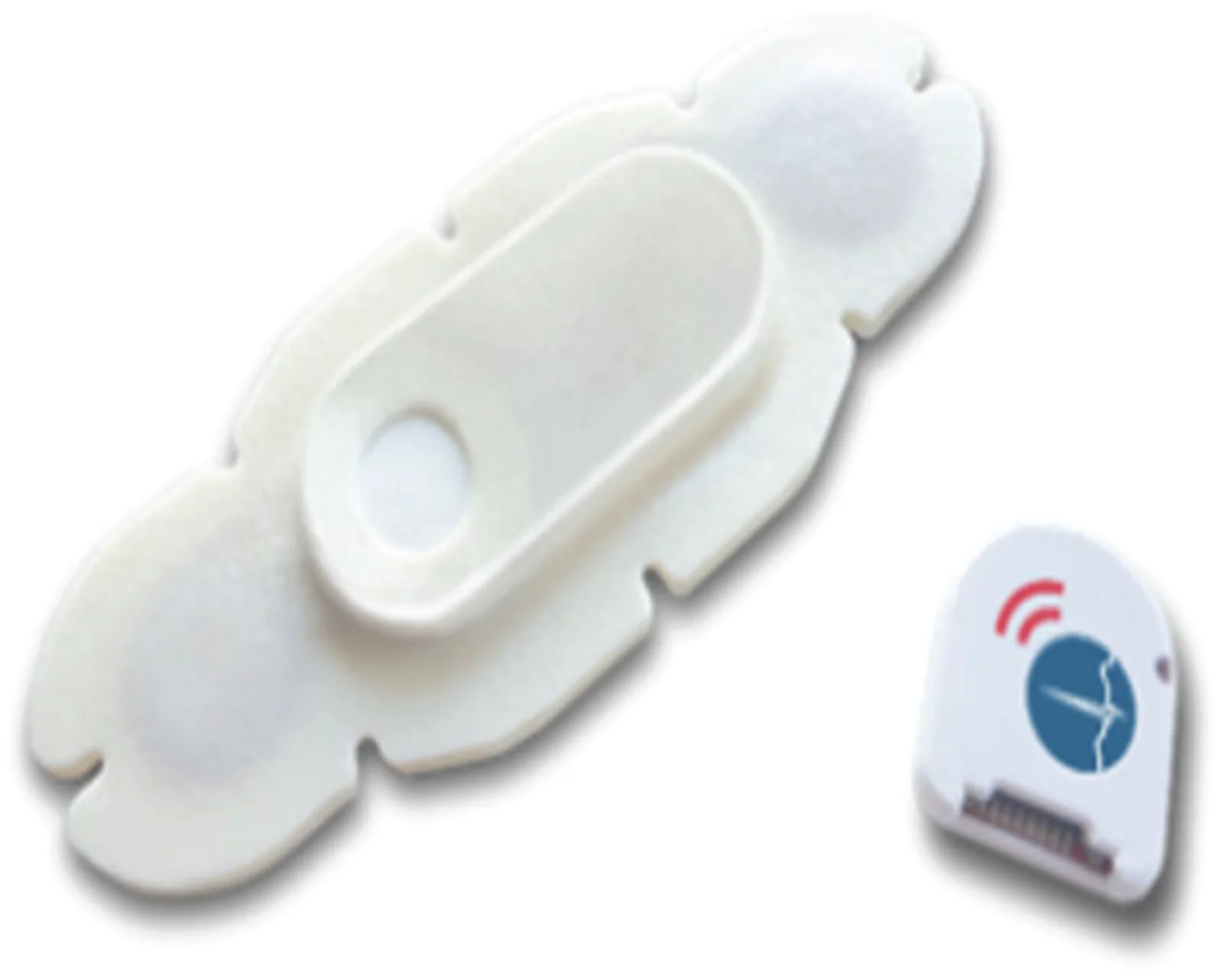
A multi-sensor monitoring device consists of a disposable sensor patch with a disposable battery and a reusable sensor electronics module. Reproduced with permission from [61]. Copyright © 2022 Mayo Foundation for Medical Education and Research. Published by Elsevier Inc. All rights reserved.

**Fig. (6):**
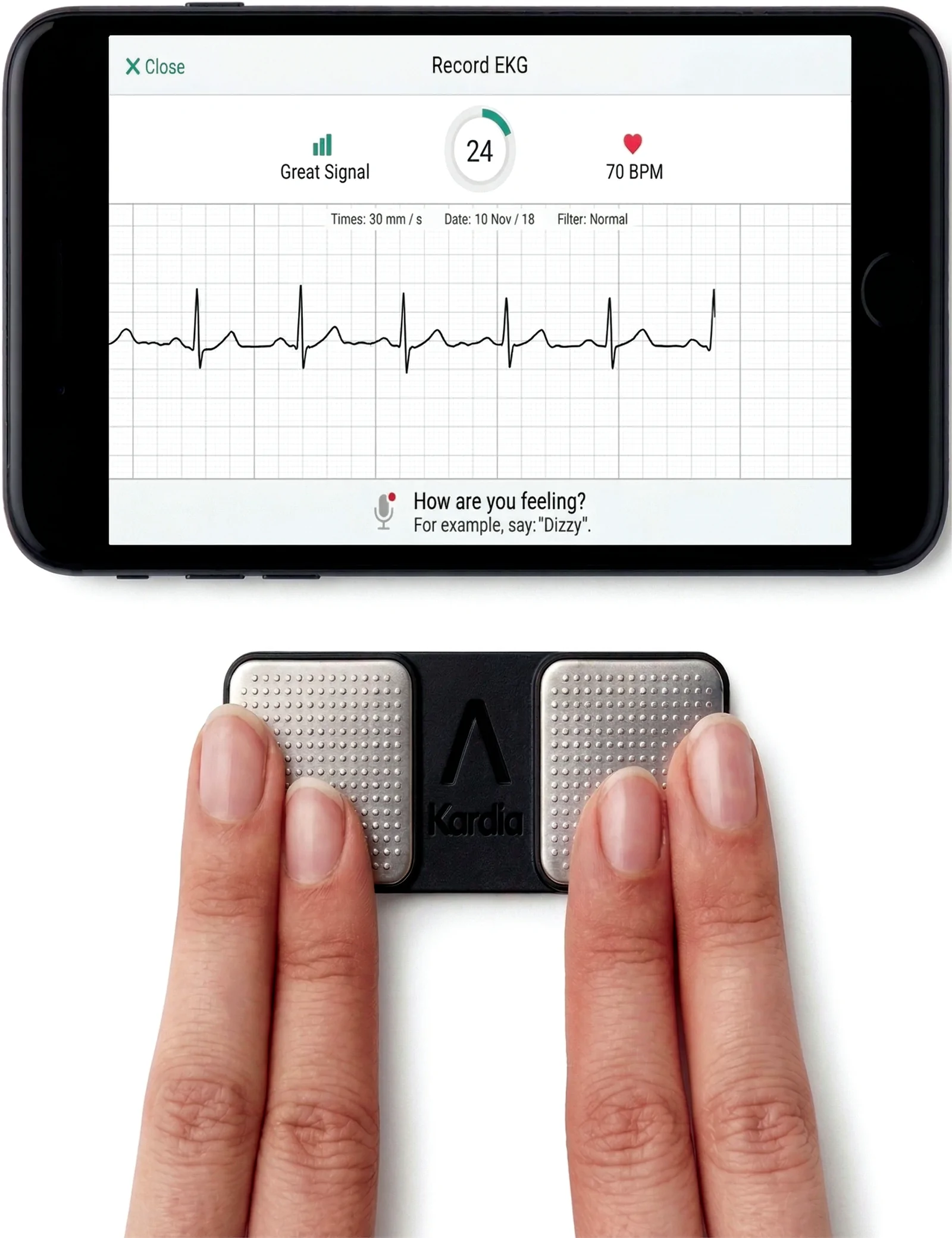
Recording an ECG with the Kardia Mobile device connected to the Kardia App. ECG: electrocardiogram. Reproduced from [2]. Under the terms of the Creative Commons Attribution License (https://creativecommons.org/licenses/by/4.0/). ©Kristina Klier, Lucas Koch, Lisa Graf, Timo Schinköthe, Annette Schmidt. Originally published in JMIR Cardio (https://cardio.jmir.org), 23.11.2023.

In 2019, a collaborative study by Stanford University and Apple found that wearable smartwatches can detect arrhythmias and atrial fibrillation and send a notification to the user, increasing the likelihood of consulting a physician. The application of ML technology can predict the 5-year survival rate of specific heart problems with an accuracy rate of 80% compared to 60% in doctors [25]. A wearable sensor that performs chronic ambulatory evaluation called MultiSENSE is programmed with a HeartLogic algorithm to alert physicians about patients with worsening HF and stratifies morbidity and mortality of decompensation by a quantifiable score [59]. Bourazana *et al*. argue that the challenges in utilizing different algorithms in AI models can create discrepancies in scoring values and parameters, *e.g.*, over-reliance on LVEF to classify HF [35]. The support vector machine used by Inbar *et al*. can identify HF patients by applying the machine learning model to the feature engineering process during Cardiopulmonary exercise tests (CPX). The model considers various factors for determining oxygen consumption and quantifies cardiac parameters, hence can identify early HF during exercise [60]. Wearable Smart watches are being developed using similar mechanisms. Artificial and convoluted neural networks can be used to assess mortality risk and HFpEF and to distinguish HF from metabolic syndrome during the CPX test [56]. Physicians could remotely monitor patients' ECGs and vitals *via* Bluetooth on their phones, which received continuous data transfer from wearable devices (Fig. **5**) on the patient's chest with Noninvasive Remote Multi-sensor Monitoring for screening of HF deterioration, as part of the LINK-HF study. However, physical activity and everyday movements raised false alarms; this study detected impending hospitalizations and decompensation in patients with HF with 85% specificity and 76-88% sensitivity comparable to an implanted device [61, 62]. The latest technological developments have equipped smartphones with photoplethysmographic imaging of jugular venous pressure and cardiac ballistics/vibrations to detect abnormal heart rhythms and HF in patients. Fundus photography, when incorporated into AI, can detect HF *via* physiological changes in the retina. FDA-certified accelerometer technology, such as the Kardia band, has been programmed into the Apple Watch to wirelessly record blood pressure and detect Atrial fibrillation in patients (Fig. **6**) [2].

Textile-based sensors used in “smart” socks are being developed to detect pedal edema and physical activity in patients. Another “smart” textile model, like the Cardioskin TM vest, can record a patient's multi-lead ECG using wearable fabric. A patch worn on the skin can record a continuous ECG for a prolonged period, unlike a classic Holter monitor, which is being developed such as the CarnationTM patch and VivaLNKTM [56]. Nahar JK *et al*. applied conversational artificial intelligence in the form of smart speakers/smart-voice-enabled technology to access HF at various stages in patients by using their voice as a biomarker. It follows the same principles of cardiac auscultation, hence alerting physicians about any deterioration, thereby improving patient care. Although preliminary research in phonocardiography showed promising results with AI, but further studies need to be done to incorporate it into clinical practice [63].

### 2.7 AI in the Detection of Biomarkers

Over the last few years, researchers have identified new types of molecules in the body that may help us better understand and manage heart failure. Two of the most promising are microRNAs (miRNAs) and long non-coding RNAs (lncRNAs). These small pieces of RNAs do not make proteins but play essential roles in how genes work. In people with heart failure, certain miRNAs, such as miR-21, miR-1, and miR-423 5p, are found in abnormal levels. These changes are linked to heart damage, inflammation, and how the heart muscle changes over time [64, 65].

The good news is that miRNAs are stable in blood, allowing them to be used in simple blood tests to assess heart health.

LnRNAs are another group of longer molecules than miRNAs and help control which genes are turned on or off. Some of them, such as LIPCAR and MHRT, are linked to worsening heart function and heart muscle changes. Because their levels often change only in certain diseases, they could help doctors understand the severity of heart failure or how it might progress [65, 66].

At the same time, technology is opening new doors in heart failure. With the help of Artificial Intelligence (AI) and telemedicine, doctors can now monitor patients from a distance. Devices like smartwatches and home heart monitors can send real-time data- such as heart rate, ECG, or even biomarker levels- to doctors. AI helps make sense of this information quickly, allowing doctors to intervene before the patient's condition worsens [64]. This is especially helpful for individuals living in areas where it is difficult to access regular medical care.

Gladding PA *et al*. studied 46 cases of decompensated HF and 20 controls in a Multiomic type of study by employing AI in ECGs and ECHOs to assess heart function. They found that AI ECG may be interchangeably used instead of the blood biomarker test called N-terminal prohormone BNP and showed increased spatial QRS-T angle at baseline. A wearable wristband detected the results of a 3D-virtual reality mental stress test; it was found that patients with HF had low alpha2 response compared to healthy individuals, thereby patients had less autonomic response governing involuntary body actions. Patients with HF also demonstrated abnormalities in the metabolic activities of mitochondria, particularly in ketone metabolism, citric acid cycle, and the kynurenine pathway. The breath acetone test is used as an HF biomarker test. Patients who are carriers of Titin gene truncation were found to have alterations in the urine Titin N-terminal Fragments associated with increased levels of NT pro-BNP biomarker levels compared to controls. AI recognized abnormal ECG in these patients; hence, machine learning and advanced algorithms can be used to accurately diagnose HfrEF [67]. Boyang C *et al*. ran six genes, which are responsible for HF with low ejection fraction, as claimed by the Gene Expression Omnibus Database, into a Random Forest classifier. They developed a diagnostic neural network model to predict the risk of each of the six genes associated with HF. Ascorin(ASPN), a secreted matrix protein and a known oncogene in colorectal cancer, may be responsible for the down-regulation of BCL2, overexpression of TGF-beta1, BAX, and phosphorylation of Smad 2 and 3 pathways to augment apoptosis in H9C2 cells of cardiomyocytes. It is also useful in preventing cardiac remodelling and fibrosis. Overexpression of Matrix remodeling-associated protein 5 (MXRA5) is related to the MAPK pathway, which is responsible for the deterioration in HF patients, and is a biomarker for calcification of aortic valves. Lumicum(LUM) promotes cardiac fibrosis and hypertrophy and is treated with osteopontin downstream signaling. Downregulation of Calponin 1 (CNN1) is responsible for poor contractility of the heart muscle and is a potential therapeutic target in treating HF patients. Glutamate-ammonia ligase/glutamate synthetase (GLUL) is responsible for endothelial function and angiogenesis. Increased levels of SERPINA3 (Serpin peptidase inhibitor Clade A member 3) alpha1 antichymotrypsin in HF patients have worse morbidity and mortality [68]. An early Screening Machine learning model (ensemble), expanded data s*et al*ong with mRMP algorithm (minimum redundancy maximum relevance) was developed to diagnose 480 patients with Hypertension LVH(>1.2 cm) and HfpEF (>50%) with vascular biomarkers, diastolic BP and ECHO ventricular volumes showing a high 92% of the Area under receiver-operating characteristic curve (ROC) hence having a high predictive performance and accuracy. Chief biomarkers that proved to have diagnostic ability were 1,3,9 matrix metalloproteinase(MMP) and tissue inhibitors of metalloproteinases (TIMP) 2,3,4, which act as biomarkers and increase, while MMP8 decreases in people with HFpEF. Biomarkers significant in HFpEF and LVH were propeptides of the NH2-terminal in procollagen type 1(PINP), PIIINP, and TIMP1-4. NT-proBNP, which was considered a gold standard, proved to be less predictive. Biomarkers like MR-proANP, Troponin, GDF-15, MR-proADM, and galectin-3 are still being researched as potential biomarkers for diagnosing HFpEF [69].

Zhang Z *et al*. ran machine learning algorithms through weighted correlation lasso regression network analysis (WGCNA) and RF model analysis. They found a positive correlation between HF and atrial fibrillation through the biomarkers GLUL, NCF2, and S100A12 (showing the strongest correlation with neutrophils in the AF group) and SRGN (tightly linked to T cells, specifically memory CD4 cells, in the AF group). In contrast, all the genes were strongly associated with neutrophils in HF patients. This study revealed a correlation between these genes and atrial fibrillation for the first time. Zhu *et al*. found NPPA, OMD, and PRELP to be diagnostic biomarkers in people suffering from dilated cardiomyopathy and HF using WGCNA and machine learning algorithms. Earlier, another study by Zhang *et al*. identified five genes, FRZB, SFRP4, ETNPPL, AQP4, and C1orf105, using transcriptome data analysis, which were demonstrated in atrial fibrillation and HF patients [70]. Siddharthan M DV *et al*. created an algorithm to study molecular markers using AI-ECGs. They found that cardiac ageing in HF patients was more advanced than their chronological age, due to lower CD34+ cells and telomerase activity. High senescence-associated secretory phenotype (SASP) proteins reduce telomerase activity and worsen HF in patients [61].

Linear regression algorithm used in Phope.py (Patients for hospital observing and predicting effects of medication as well as exercise) used by over 5668 users globally is a biomarker predictive model taking into consideration 5 data inputs: test date, HBA1C (diabetic biomarker), NT-proBNP (HF biomarker), degree 1 (polygonal regression for diabetic biomarker) and degree 2 (polygonal regression for HF biomarker) can detect the risk of diabetes and HF [56].

### 2.8 Deep Learning as a Diagnostic Tool

In A DL model for HF mortality prediction, Li (*et al*.) described a novel approach to identifying and improving heart failure outcomes by using the deep learning method, Multi-head Self-attention Mechanism (DLA-MSM), to predict Heart failure mortality in patients within 30 days, 180 days, 1 year, and two or more years. The training strategy consisted of a 5-fold cross-validation to ensure an appropriate capture of significant trends. The dataset was then divided into training, validation, and testing sets and trained for 120 epochs using the RMSprop optimizer on the training dataset. Performance was based on accuracy, positive predictive value, negative predictive value, recall, F1 score, and Area under the curve (AUC). The datasets tested were the W30D dataset, W180D Dataset, W365D dataset, and A365D dataset. WD30D offered the best performance, with model stabilization achieved at 80 epochs. In this dataset, the Area under the positive rate was 91%; the accuracy was maintained at 84.56%, while the F1 score rose from 23.01% to 78.69%, allowing the model to distinguish between alive and dead patients and demonstrating satisfactory precision and recall. W180D dataset comparably had a lower stability of 82.05%. This increased imbalance rate resulted in an f1 value of 56.55% and an AUC value of 82.08%. The V365D dataset had a higher imbalance rate, resulting in a more significant volatile loss. AUC decreased to 75.00%, with a micro average of 91% and a macro average of 75.00%, indicating comparably higher instability and difficulty in comprehending the given data. The A365D dataset indicated a 75.07% accuracy, F1 of 46.34%, and a stable Area under a positive rate of 93%. This dataset identified patients who expired after 1 year, but only 66.19% of the cases died after two or more years of the statistical period. The dataset demonstrated a similarity in characteristics between patients who die after 1 year and those with a longer lifespan. Hence, this dataset offered a more accurate prediction of mortality. For the F1 score, improvements were observed in W180D, W365D, and A365D, indicating better comprehension of imbalanced datasets by DLS-MSM. DLS-MSM effectively identified deceased patients most accurately overall, offering a consistent 99.88% negative prediction value [71]. Table **1** shows AI-based diagnostic modalities in heart failure, highlighting key advantages and disadvantages.

## 3 TREATMENT OF HEART FAILURE USING AI

A systematic review by Błaziak *et al*. (2022) revealed an increasing number of Artificial Intelligence (AI) implementations in heart failure management, significantly non-linear Machine Learning Models, due to the added complexity that increases accuracy. However, this is accompanied by poor external validation for an independent dataset. Additionally, machine learning predictive models outperformed standard statistical models in terms of external validation, enabling more individualized, patient-level management [72]. Fig. (**7**) illustrates the comparison between treatment models of conventional v/s AI approaches for the treatment of HF.

Another example of AI implementation is a Japanese study that utilized a Machine Learning (ML) predictive model derived from administrative claim data (ACD) and the Japanese Registry of Acute Decompensated Heart Failure (JROADHF). This study showed that the performance of the simple ML model called the Simple Model by Artificial Intelligence for HF risk stratification (SMART-HF) was equal or superior to those of the standard risk models with a C-statistic of 0.765 [95% confidence interval 0.739–0.7^91^] and a Brier score of 0.124. This new model requires a few data inputs, which can be obtained with a brief interview, even by inexperienced personnel [73].

Furthermore, Machine Learning models can be used for purposes other than risk stratification, as demonstrated by a study by Jing *et al*., which used data from an EHR system that used 276,819 clinical episodes from nearly 27,000 patients to predict all-cause mortality after one year. Clinically relevant evidence-based approaches, both diagnostic and therapeutic, known in this study as “care gaps,” can also assist with creating new value-based payment models [74].

For a variety of reasons, including access issues, clinician under-prescription, or medication intolerance, most patients with heart failure do not obtain the recommended dosages of evidence-based medical therapy. By evaluating heterogeneity in the response to Heart Failure (HF) medications, 41 machine learning algorithms were applied to enhance the medical management of HF [75]. For instance, ML techniques were used to examine heterogeneity in HF medication response across propensity-matched clusters using the electronic health records of 44,886 individuals in the Swedish Heart Registry. Thirty individuals had notable differences in 1-year survival and response to treatments; four clusters were established based on demographic, NYHA class, LVEF, comorbidities, and lab indices. As a result, ML may be utilized to divide HF patients into high- and low-risk subgroups more accurately and determine which ones are most likely to benefit with the least side effects in GDMT [75].

AI has enhanced medical treatment through medications, innovative cardiovascular pharmacological therapies for managing hypertension, and a novel method of cardiovascular risk stratification and heart failure phenotyping [74]. Shameer *et al*., employed the concepts of precision medicine by developing a novel classification system for HFpEF in an attempt to better explain the pathophysiology of heart failure. The new classification involved the process called phenotyping, wherein AI-based unsupervised deep learning algorithms analyzed all relevant patient data on extensive clinical, laboratory, echocardiogram, and imaging studies [76].

Policies guiding the translation of AI technologies into practice will likely continue to evolve as these technologies advance and attain greater therapeutic value [77]. Access for patients will facilitate the broader use of implantable technology and remote monitoring systems, particularly in more vulnerable populations. Inequity in access to these technologies should be addressed so that no patient with heart failure is deprived of equitable treatment. Results in health data collection and transfer, particularly for AI applications or remote monitoring, create data security and patient privacy challenges. This calls for stringent procedures for data protection in healthcare systems to ensure the protection of patient information. Such changes are imperative in healthcare workflow and practice so that AI applications and device-based interventions will be integrated into clinical practice. This may involve training health workers, developing standard operating procedures, and establishing clear pathways for action based on monitoring data [77, 78].

Artificial Intelligence has a significant role in optimizing existing therapies. Sotalol and dofetilide are frequently used to treat atrial fibrillation (AF). These medications cause QT prolongation because they prolong the repolarization phase, which is how the myocardium experiences its antiarrhythmic action. When these medications are administered, especially dofetilide, associated risks include significant QT prolongation and potentially fatal cardiac pro-arrhythmia, which mandate intensive patient monitoring with a continuous ECG in a hospital setting. Dose adjustment may be indicated in cases of marked QT prolongation, concurrent drug therapy known to prolong the QT interval, and changes in renal function, as the kidneys are the primary organs responsible for metabolizing both sotalol and dofetilide. All these factors increase the indication for periodic QT interval checks during long-term treatment with these drugs. Using serial and consecutive 12-lead electrocardiograms and associated data on plasma dofetilide concentration, for instance, in 42 patients receiving either dofetilide or placebo in a crossover randomized clinical study, plasma dofetilide concentration could be predicted by a deep learning method with an excellent correlation r = 0.85. By contrast, a linear model of the corrected QT interval, with a coefficient of 0.64, was associated with dofetilide concentrations [79]. According to this research, the QT interval may exaggerate or underestimate the proarrhythmic risk in certain patients based on an inaccurate reflection of their plasma dofetilide content. The optimal dosing schedule for dofetilide therapy utilizes machine-learning techniques, including guided, self-directed, and adaptive learning [79].

## 4 AI IN PROGNOSIS AND RISK STRATIFICATION OF HEART FAILURE

### 4.1 Predictive Models - Patient Outcomes, Mortality Rate, and Readmission Rates

The integration of artificial intelligence in forecasting heart failure outcomes, mortality, and readmission rates has seen substantial advancements. For instance, the Mayo Clinic developed a predictive model utilizing AI to enhance electrocardiograms (ECGs). This innovation achieved a 93% accuracy rate in detecting left ventricular dysfunction, facilitating early diagnosis and improved management of heart failure risk. Similarly, Google Health utilized deep learning techniques in its AI imaging tool, enabling the early detection of heart conditions and enhancing the accuracy of heart failure diagnoses [1].

The XgBoost algorithm applied machine learning to key biomarkers, predicting heart failure following an acute myocardial infarction and enhancing early warning and intervention efforts [80]. These AI and machine learning models have significantly advanced the prediction and management of heart failure, improving patient care and outcomes.

### 4.2 Key Algorithms and their Accuracy

Several algorithms have proven essential in predicting heart failure outcomes, mortality rates, and readmissions, each excelling in handling medical data. For example, Random Forests construct multiple decision trees and combine their classifications, enhancing accuracy and robustness, particularly in managing missing data [81]. Support Vector Machines (SVMs) identify the optimal hyperplane for data categorization, demonstrating high accuracy in binary classification tasks and excelling in high-dimensional data analysis when the appropriate kernel is used [82].

Gradient Boosting Machines (GBMs) sequentially build models, with each model correcting errors from the previous one, resulting in high predictive accuracy and versatility across various data types [80]. Recurrent Neural Networks (RNNs) were adept at recognizing patterns in sequential data, making them ideal for time series predictions by retaining information from previous inputs. Their ability to capture temporal dependencies was particularly valuable in heart failure progression prediction [81]. Long Short-term memory networks (LSTMs), a subtype of RNN, improved on standard RNNs by learning long-term dependencies more effectively, making them highly accurate for time series data [1]. Finally, autoencoders, a type of neural network used for unsupervised learning, compresses data into lower-dimensional representations and then reconstructs it, thereby improving the performance of other predictive models by facilitating dimensionality reduction and feature extraction [80].

Additionally, in patients with HFpEF, AI-enabled ECG analysis can identify the presence of left atrial myopathy in patients with HFpEF, which is associated with an increased risk of atrial fibrillation and long-term mortality [83].

AI algorithms can predict adverse outcomes in patients with HFpEF, such as mortality and hospitalization for heart failure. These models can integrate large amounts of data to provide insights that help clinicians make more timely and accurate decisions [84].

With their diverse strengths, these algorithms have collectively enhanced the precision of heart failure predictions, thereby contributing to more effective patient management.

### 4.3 Risk Stratification

The implementation of artificial intelligence in heart failure to stratify risk is a method that correctly identifies adverse effects, hospitalizations, and even death due to the precision of the data provided daily. The analysis of the data used in the long term demonstrates that artificial intelligence is an ally to improve clinical judgment and the accurate diagnosis of heart failure due to the ability to implement a large amount of information and adapt it to the patient, thus achieving an adequate stratification [25].

Artificial Intelligence is a tool available to everyone. People are increasingly looking for ways to determine their risk of developing a disease that can lead to chronic dependence. Concerning heart failure, we see patients who present acutely without having a clear view of the outlook that awaits them. For this reason, algorithms have been developed using images such as echocardiograms performed in asymptomatic patients with risk factors, which, through machine learning, predict the incidence of heart failure [85].

In recent years, we have evaluated the need to make continuous adjustments in treating multiple diseases, reducing “therapeutic inertia” over time. Now, artificial intelligence provides the ability to optimize medication prescription, achieve adequate doses, avoid adverse effects, and consider drug interactions. In this way, the objectives are achieved [86].

The participation of patients in their own disease treatment is becoming increasingly necessary, which is why their active involvement is crucial when determining risk. Various mechanisms were implemented to enable interaction through artificial intelligence based on the symptoms and variables presented, resulting in a decrease in cases of disease exacerbation [87]. Telemedicine associated with artificial intelligence through learning machines can detect minimal physiological variables in real-time, helping to improve medical performance. A project called Digital Twin has the aim to create a person with the same characteristics and with a great amount of information, which will determine which personalized treatments will have the best results [88].

All of this causes an increase in expectations when stratifying the patient's risk during the natural history of the disease. Although there is still a lack of more precise data and significant studies to be carried out, it provides a clearer picture.

### 4.4 Impact on Clinical Decision-making and Resource Allocation

Artificial Intelligence is an extension of our cognitive abilities, allowing us to redesign processes and inform decision-making in healthcare. The allocation of resources in heart failure aims to distribute healthcare resources, reducing total healthcare costs appropriately. Heart failure represents a high cost in all countries due to its prevalence and high mortality rate worldwide [89].

**Fig. (8):**
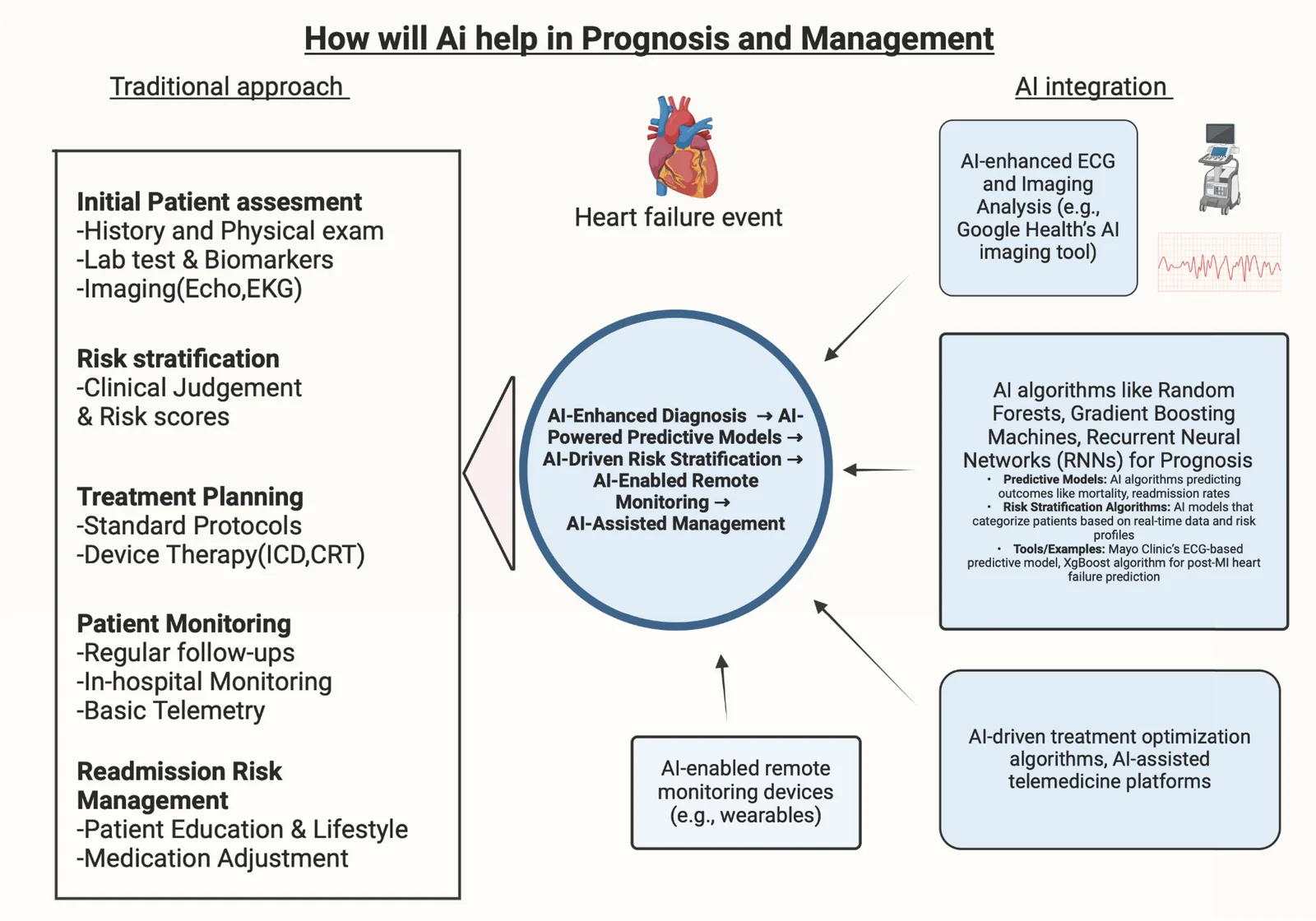
Approach of AI to help in the prognosis and management of heart failure patients.

Over the last few years, multiple researchers have made significant efforts to allocate resources effectively. For this reason, improvement strategies were implemented in the preventive, diagnostic, and therapeutic approaches in which the patient, doctor, and artificial intelligence participate, reducing the risk of complications [90]. Some examples of this would be:

The electrocardiogram is a readily accessible tool in all healthcare services. Through multiple algorithms, artificial intelligence can determine the risk of developing heart failure (Fig. **8**) [38, 42]. Even in issues as crucial as rehospitalization, machine learning can determine the risk of acute heart failure decompensation. Through continuous monitoring, the LINK-HF study presented interesting results for early medical intervention [62].

Treatment options are not left out of the wide range of proposals by artificial intelligence, which is one of the novelties that provide personalized management and efficient resource utilization, even in situations where patients do not require face-to-face medical consultations, as seen in telemedicine [57]. Implementation of the systems and their maintenance, including the entry of information and sustaining the database, are costly, and the investment requires multiple agencies [91].

## 5 AI IN HEART FAILURE PATIENTS- PROGNOSIS AND MANAGEMENT

Artificial intelligence (AI) is reshaping healthcare by tracking and controlling persistent situations, including heart failure. Integrating AI with wearable devices, telemedicine platforms, and advanced record analytics enables continuous patient monitoring, early detection of complications, and timely interventions, all of which are essential for improving patient outcomes. AI-powered wearable devices play an essential role by constantly gathering real-time records on important characteristics such as oxygen saturation, heart rate, blood pressure, and breathing rate. One implantable sensor that tracks pulmonary artery pressure is the CardioMEMS HF System, which transmits these records to healthcare providers, facilitating early intervention [87]. Similarly, the Zio patch, through iRhythm, uses AI to constantly monitor heart rhythms and detect arrhythmias related to heart failure. AI models analyze electrocardiogram (ECG) records to understand styles indicative of deteriorating heart function. At the same time, smartwatches with AI-enabled sensors are being increasingly incorporated into heart failure care, thereby enhancing adherence to fitness guidelines among affected individuals [91, 92].

AI-driven remote tracking offers numerous advantages for managing heart failure. Early detection of physiological modifications enables faster interventions, reducing health facility readmissions, and improving long-term outcomes. AI structures can identify subtle modifications in a person's circumstances that are likely overlooked by traditional methods, thereby helping to prevent disease progression [92]. AI algorithms can tailor care primarily based on a patient's physiological data, resulting in custom-designed treatment plans and more suitable patient engagement. Patients monitored remotely using AI-guided devices frequently report better engagement ranges due to continuous feedback, reminders, and actionable insights, which enhance the management of chronic conditions like heart failure [93]. Moreover, AI-based remote tracking systems are cost-effective, reducing the need for frequent in-person visits and hospitalizations [94]. Predictive analytics, a key characteristic of AI, plays a significant role in identifying early signs of deterioration, enabling healthcare providers to adjust treatments directly and enhance patient care [91].

Telemedicine, which has become increasingly important in managing heart failure, is also gaining prominence through the use of AI. AI-powered selection helps examine patient-reported signs during teleconsultations and provide customized treatment recommendations based on clinical guidelines [95]. Virtual assistants and AI-driven chatbots enhance patient engagement through tracking symptoms, presenting treatment reminders, and answering queries, which allows healthcare providers to focus on higher-level decision-making. Integration with digital fitness record (EHR) structures ensures that telemedicine interactions are data-driven, with tips informed through the state-of-the-art patient records and tracking data [62]. Predictive models within telemedicine systems aid in prioritizing patients requiring urgent care by analyzing real-time data, which is particularly beneficial for managing large populations of heart failure patients [96]. Combining AI and telemedicine enables the achievement of greater accuracy, data-driven care, and efficiency [97].

Successful telemedicine programs have effectively included AI in heart failure management. For example, the Heart Failure Telehealth Program at Cleveland Clinic utilizes AI to analyze information from wearable devices, resulting in fewer hospital readmissions and improved patient outcomes [57]. The Tele Heart initiative leverages AI to predict heart failure exacerbations by analyzing remote tracking statistics, resulting in timely interventions and improved adherence to treatment [98]. Similarly, the Stanford AI-based Remote Monitoring Program utilizes AI analytics to predict decompensations, decrease emergency department visits, and promote proactive care [99]. These examples illustrate AI's transformative impact on continuous sickness control and its ability to enhance contemporary healthcare.

AI has significantly advanced patient tracking and management, particularly in the care of patients with heart failure. Table **2** presents the characteristics of AI related to heart failure. It accurately describes the advantages and disadvantages.

## 6 DISCUSSION

The risk of heart failure has increased in the last 25 years, according to the Framingham Heart study cohort, and the US population has a lifetime risk of 24%. Therefore, there is a need for advanced technology like the implementation of artificial intelligence into routine diagnostic modalities like ECGs, ECHOs, Cardiac MRI, and wearable sensors to equip physicians with early screening tools to diagnose and predict heart failure to provide early intervention. Applications of AI in daily clinical practice and even in hospital settings have reduced the labour burden on physicians, making it simple to perform early screening and detection of heart failure or early stages of decompensation with ease. In remote settings, AI algorithms detect early HF from basic imaging modalities like X rays or ECGs and prompt patients to visit a specialist for early treatment. Physicians can monitor patients in real-time using active or passive tele-monitoring devices equipped with wearable sensors and mobile apps, which utilize AI to detect early signs of decompensation. Patients receive alerts to visit hospitals for consultation, thereby decreasing morbidity and mortality. AI algorithms used to analyze biomarkers in HF patients can prove to be an effective screening tool even before the use of sophisticated imaging technology, as they have a high negative predictive power. In a few years, the applications of AI will be incorporated globally in the everyday clinical practice of cardiologists for screening, classifying, and diagnosing various heart failures with varying ejection fractions and other cardiovascular diseases. Table **3** represents AI methods in heart failure diagnosis, management, and prognosis.

In recent years, AI models for managing heart failure have been developed using patient data collected through registries and electronic systems, enabling their application to patients outside the original data set and demonstrating their practical utility.

This method has been quite effective except for minor limitations. AI models are also used for monitoring drugs during heart failure to minimize possible side effects and assist with dosing adjustments. The most apparent risk of such models is patient privacy, as this emerging technology will skip confessional security borders for data monitoring and exchange. Promoting awareness among healthcare providers and enacting policies to guard against this risk is necessary.

The application of Artificial Intelligence (AI) in forecasting heart failure outcomes, including mortality rates and hospital readmissions, has significantly enhanced patient care. For instance, the Mayo Clinic has developed an AI system that enhances the accuracy of identifying heart problems using electrocardiograms (ECGs), achieving a 93% success rate. Likewise, Google's AI imaging technology utilizes deep learning to detect heart conditions early, allowing the doctors to manage patients more effectively.

Other models of artificial intelligence, such as the ESCAPE Risk Model and the Heart Failure Survival Score (HFSS), interpret clinical information in order to evaluate risks in patients and tailor treatment regimens more precisely. Sophisticated instruments such as the Signal IHF and the XgBoost algorithm utilize cardiac devices and key biomarkers to forecast heart failure events and issue early warning signals, which can lower hospitalization rates and enhance survival. Random Forests, Support Vector Machines (SVMs), and Gradient Boosting Machines (GBMs) are crucial in handling complex medical data, allowing for better prediction of patient outcomes.

These technologies assist physicians in making informed decisions and managing resources more efficiently in heart failure treatment.

**Fig. (9):**
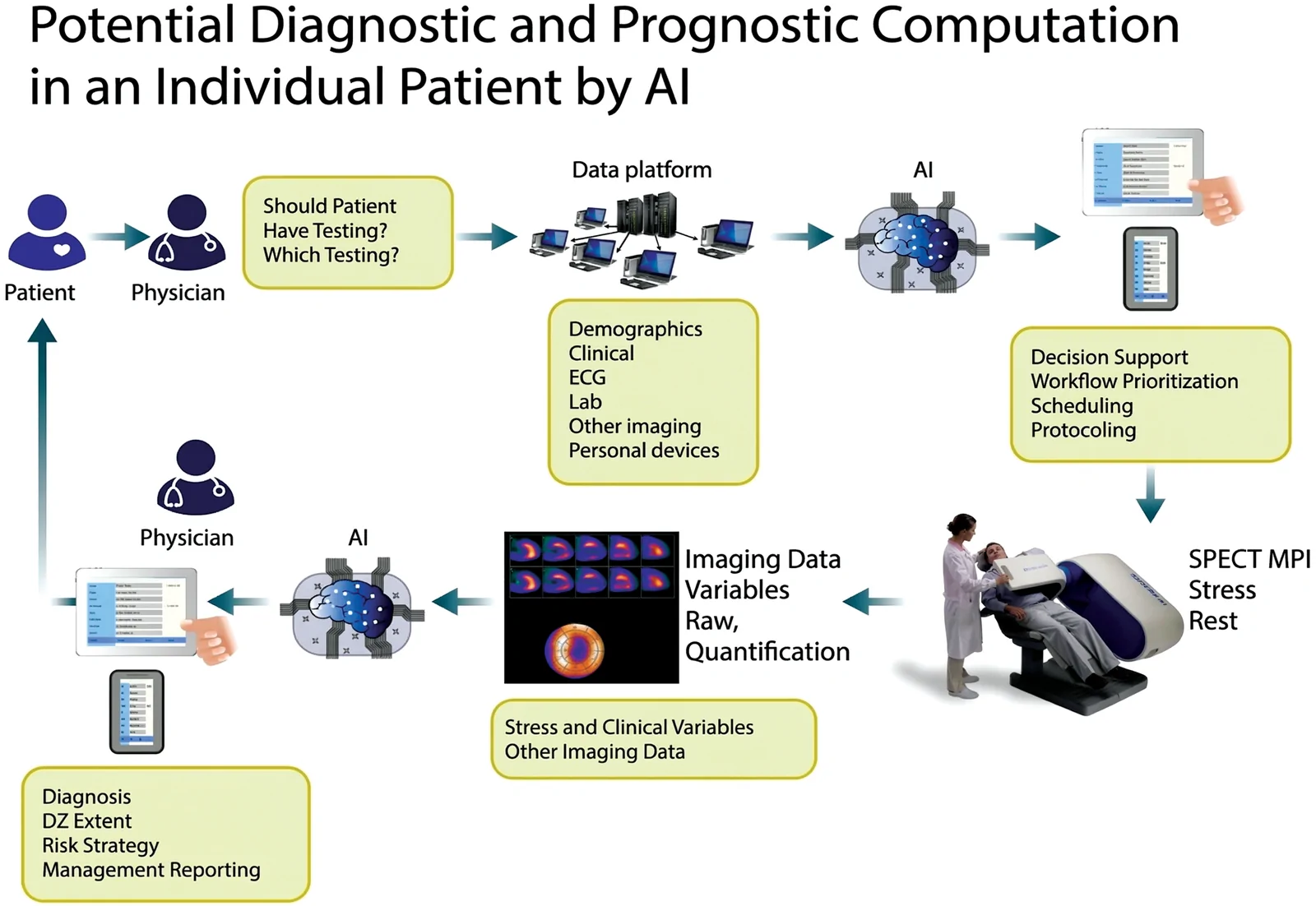
Artificial intelligence (AI) algorithms can be utilized in nuclear cardiology in various ways, including enhancing diagnosis and risk stratification (strat), patient-specific management, and reporting, as well as aiding in clinical decision-making regarding test appropriateness, test selection, scheduling, workflow prioritization, and protocol development. The disease is denoted by the letters DZ, ECG, Lab, MPI, and SPECT, which denote electrocardiography, laboratory tests, and single-photon emission computed tomography, respectively. Reproduced with permission from [1]. Copyright (2020), Mayo Foundation for Medical Education and Research.

Table **4** represents the comparative performance of ai models in heart failure diagnosis. Aside from enhancing and expanding research to improve diagnosis and prognosis, future AI work in nuclear cardiology must be directed toward developing AI-based algorithms to assist in clinical decision-making including test appropriateness, test selection, scheduling, workflow prioritization, protocoling, reporting, and patient management (Fig. **9**) [1].

A promising tool for a noninvasive, radiation-free thoracic morphometric assessment involves the use of innovative anatomical biomarkers, such as the Modified Haller Index (MHI), which is based on echocardiographic and external chest measurements. It presents significant opportunities to enhance AI-driven models of cardiovascular risk prediction. By spotting subtle changes in chest wall conformation, the Modified Haller Index (MHI) departs from the conventional Haller Index. Especially in women with small body surface area (BSA) and low cardiovascular risk, an MHI greater than 2.5 indicates a concave chest shape, which correlates with a reduced prevalence of obstructive coronary artery disease (CAD), fewer cardiovascular events, and improved exercise tolerance. The results show that thoracic anatomy affects coronary flow dynamics and myocardial stress response during exercise. Incorporating MHI into AI systems using wearable sensors, ECG, Exercise stress echocardiography (ESE), and EHR data could improve tailored risk stratification, reduce false positives in stress imaging (especially in cases of Mitral valve prolapse or left ventricular dyssynchrony), and minimize unnecessary testing in low-risk populations. Within tailored treatment plans, MHI could improve the interpretation of EHRs and wearable sensor data. MHI-integrated future artificial intelligence models could offer anatomically informed, reasonably priced screening techniques, enabling early prevention and precision cardiology in local communities [100-102].

Such developments will not eliminate physicians and other healthcare professionals; they will provide them with extremely accurate devices to diagnose diseases, more reliably classify risks, and enhance patient-specific care.

In summary, AI plays a crucial role in improving the prediction of heart failure outcomes, optimizing treatment, and reducing healthcare costs, leading to more personalized and effective patient care.

## 7 LIMITATIONS AND PROSPECTS

Limitations of AI-driven clinical decision systems (AI-CDS) can be categorized into two main groups: knowledge variability between physicians and limitations in the performance of Artificial Intelligence models.

The level of expertise between one physician and another differs significantly depending on their experience using AI models and their ability to interpret data obtained from AI analysis. This variability can alter the usefulness of AI [58]. Deep learning and Machine learning models are the most used algorithms in AI-CDS and have shown very good outcomes in helping clinical experts maximize the assessment of heart failure. However, the performance of these algorithms can be altered due to inaccurate training data-these defects in model training impact AI's diagnostic, predictive, and analytic capacities [57].

The main challenges for optimizing AI support in decision-making regarding heart failure management include training physicians in the correct use of AI models, the collection of accurate data, and the evaluation of results given by AI. Additionally, it is crucial to prioritize the correct data training of AI models, as their performance and effectiveness greatly depend on the quality of the available data [109]. The disproportionate use of AI can widen the healthcare gap between lower socioeconomic and elite class of the population. AI has been shown to amplify the inherent bias in racial discrimination due to the missing data from minorities in databases. Careful analysis is needed to eradicate the implanted prejudice and work toward a truly representative cross-section of the community [109].

Sun, X, *et al*., concluded that most studies have not clearly explained how AI models can detect diseases due to a human lack of comprehension of AI networks [24]. Another major problem is that the existing results are based on limited research sites and their specific population [24]. Prospects regarding AI support systems include improved wearables, digital medicine, and a better understanding of AI models to develop individualized treatment options and disease prevention [33]. Stehlik *et al*. analyzed wearable monitors to predict heart failure hospitalizations and shared the limitations of the observational study. The data set was not homogeneous and included more male patients. Other factors, such as compliance with sensor use and the incompleteness of data generation and assessment, can influence the interpretation and conclusions drawn. Therefore, more studies and extensive controlled trials are needed to generate generalizable results [62].

The reliability of AI models is affected by dataset size constraints, as smaller datasets may lead to overfitting and reduced model robustness [104]. The capacity for generalization of results remains a concern, as variations in data collection methods, imaging techniques, and patient characteristics affect the applicability of AI findings to broader clinical use [105] and selection bias limits the generalizability of many research studies because models are frequently trained on particular datasets that may not be entirely representative of other patient populations [103]. Despite these limitations, the future of artificial intelligence in clinical support systems for medical care is exciting. The application of new technologies provides us with better opportunities to improve the management of chronic diseases, such as Heart Failure, and modernize patient care in the cardiology setting.

## CONCLUSION

By utilizing complex AI systems, such as machine learning and deep learning, healthcare professionals can considerably amplify diagnostic accuracy, personalize treatment plans, decrease healthcare costs, and enhance patient outcomes. For example, AI-driven electrocardiogram analysis enables the early detection of HF-associated electrocardiographic features that may be missed or overlooked by physicians. Additionally, newer AI algorithms can help render more precise and nuanced interpretations of echocardiograms, MRIs, and other imaging modalities. Another application of AI is the stratification of patients with high risk, allowing for preventative interventions that can reduce hospitalizations and rates of mortality correlated with heart failure. Nevertheless, the effective implementation of AI in the clinical domain faces several obstacles, including ethical considerations, data privacy issues, and the necessity for rigorous verification of AI models to ascertain reliability, reproducibility, and safety covering heterogeneous groups of patients. Subsequent research should focus on further developing these algorithms, expanding their usability across diverse patient populations by considering additional variables such as age and race, and integrating AI assistants into existing healthcare processes. As the landscape of heart failure management continues to shift, AI's potential to improve patient care and optimize health outcomes is undeniable. AI is emerging as a pivotal pillar in the future management of heart failure.

## AUTHORS’ CONTRIBUTIONS

The authors confirm their contribution to the paper as follows: study conception and design: VD, SB and JA; data collection: EA, FA, PC and MY; data analysis or interpretation: FA, PC, SB and MY; methodology: VD, EA, SB and MY; investigation: AY, MN, MT and JS; draft manuscript: FA, PC, JA, EA, JS, SB, MY, GY and PS; critical revision of the manuscript for important intellectual content: VD, JA and SB; supervision: VD. All authors reviewed the results and approved the final version of the manuscript.

## Figures and Tables

**Fig. (2):**
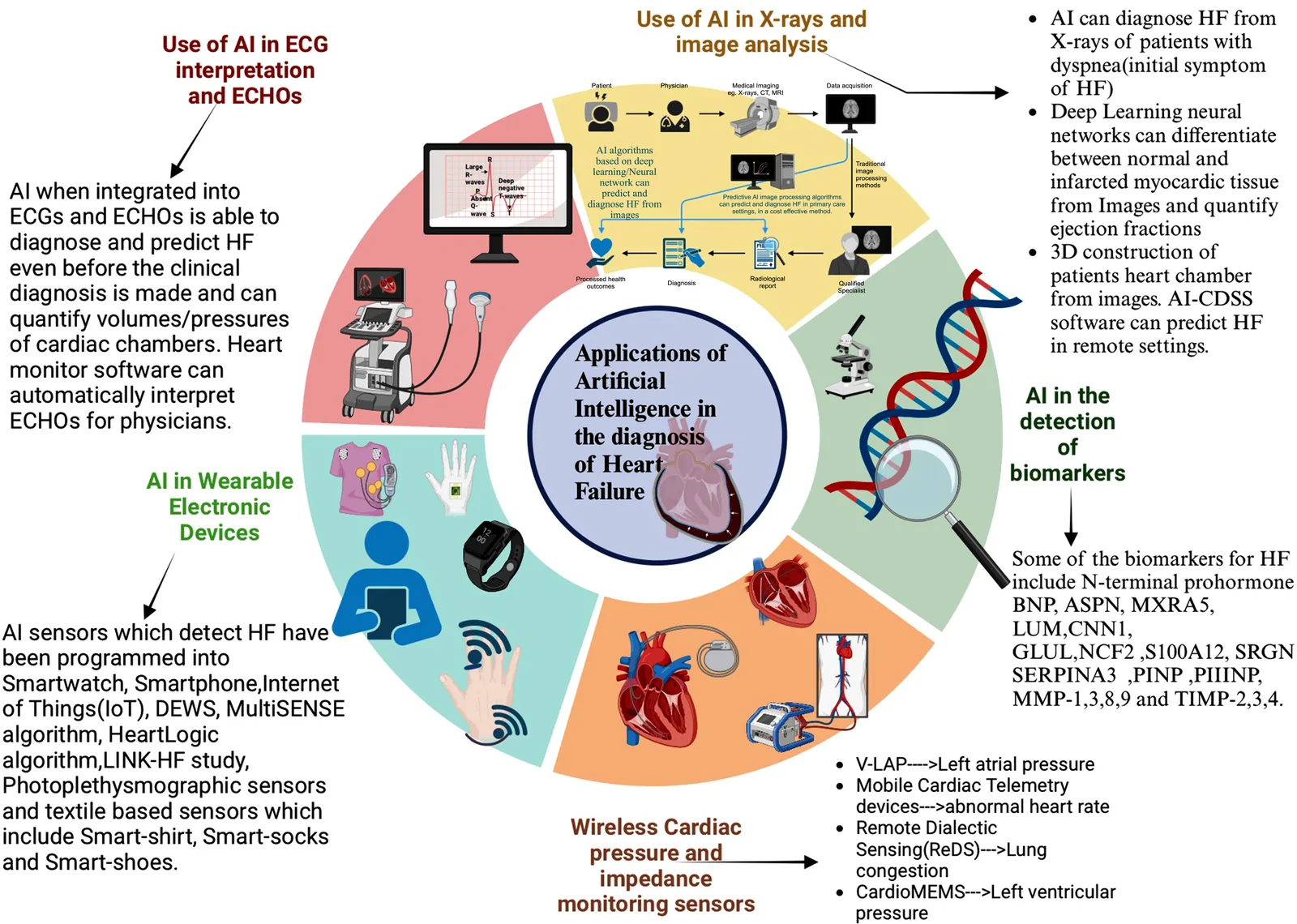
Different avenues in which artificial intelligence has served as a diagnostic tool to aid in HF diagnosis (Created with BioRender.com).

**Fig. (3):**
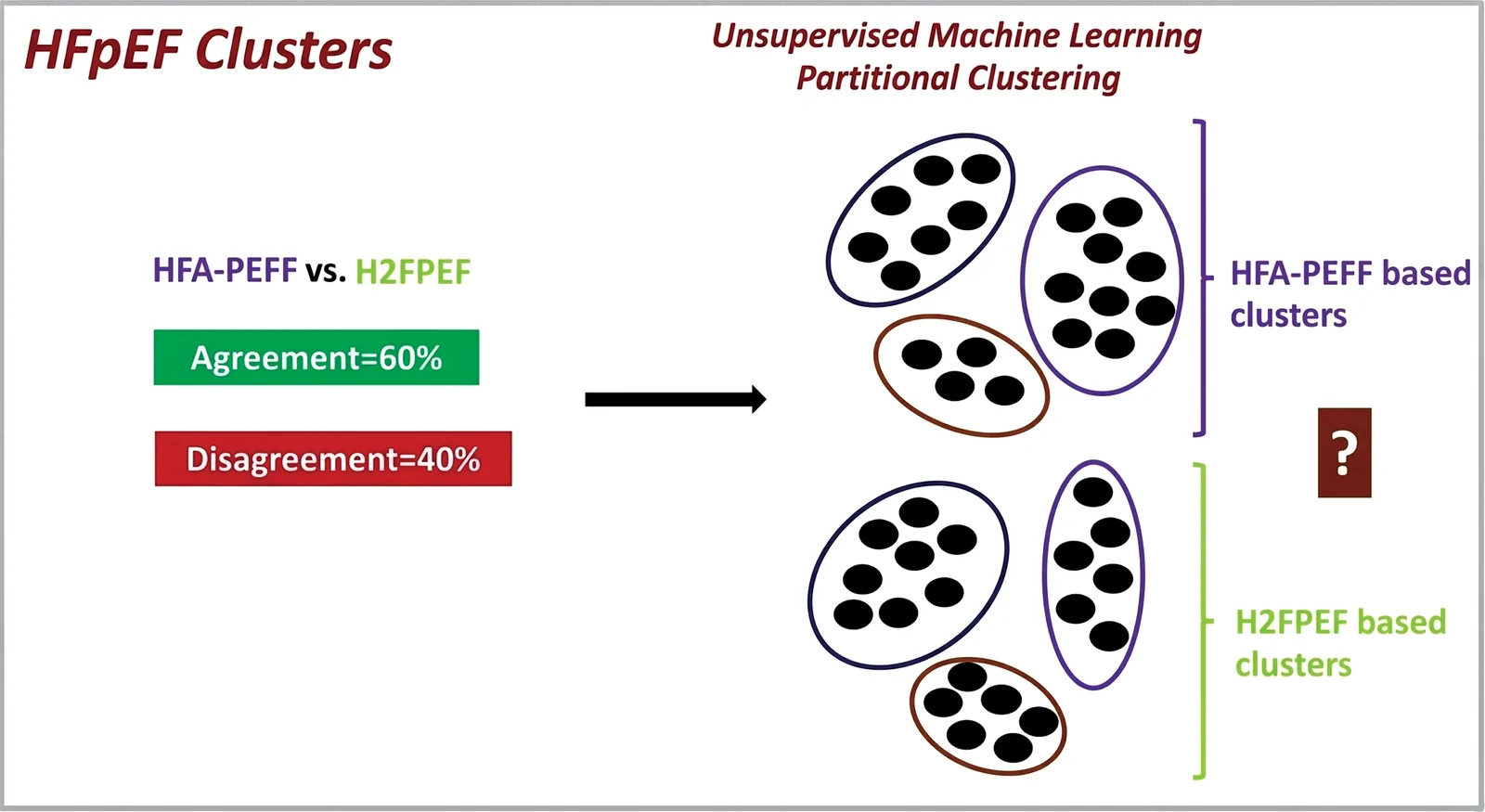
Reproduced under creative commons license [35].

**Fig. (7):**
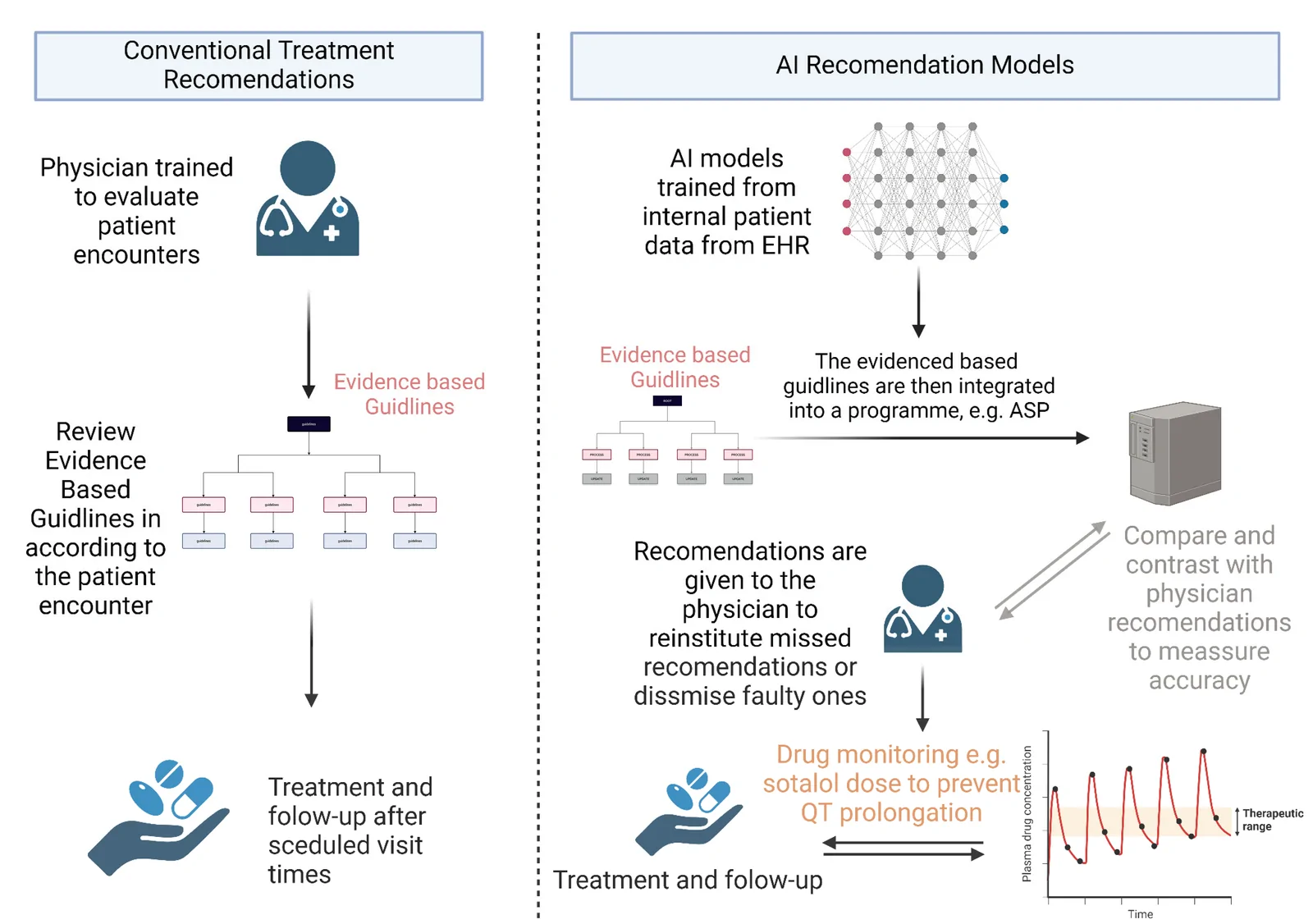
Compares the methodologies used between conventional *vs.* AI in the treatment of HF (Created with BioRender.com).

**Table 1:** Overview of AI-based diagnostic modalities in heart failure, highlighting key advantages and disadvantages.

**Diagnosis of HF using AI Methods**	**References**	**Advantages**	**Disadvantages**
AI-based ECG *vs*. Blood Biomarker (NT-proBNP)	[67]	It can be used in place of conventional blood indicators to diagnose heart failure. Increases the spatial QRS-A2T angle detection.	Perhaps not as accurate as more thorough blood testing in capturing all the intricacies of HF.
Deep Learning for Ejection Fraction Prediction	[33, 35]	DL models, such as CNNs, enhance the accuracy of ejection fraction measurement, automate diagnostic work, provide accurate and replicable results, deal with cardiac service in underprivileged areas, and improve the model's capability to predict the prognosis of heart failure.	Challenges such as reliance on high-quality data, limited generalizability across different populations, lack of interpretability, high computational costs, and potential for misdiagnosis emphasize the need for careful integration and human oversight.
Wearable Technology for Autonomic Response Monitoring	[25, 46, 56, 62]	Real-time telemonitoring for early warning systems with signal access in diagnosing arrhythmias and HF.	False alarms, anxiety due to alarms, added information that leads to information overload, cost, privacy concerns, and regulatory testing.

**Table 2:** Characteristics of AI associated with HF.

**Aspect**	**Details**	**Study Algorithms Used**	**Key Example**	**Performance/Outcomes**	**Challenges/Limitations**
AI in Heart Failure Management	There is an increasing use of non-linear Machine Learning models and AI for personalized heart failure management.	Machine learning, answer set programming	[72]	Improved accuracy, but with external validation issues.	Lack of external validation, practice style differences. The available financial resources are still insufficient to implement the models.
AI in HF Risk Stratification	Risk stratification can be facilitated with the aid of AI models that access and analyze clinical data. Risk can be determined with low-cost imaging.	Random forests, recurrent neural networks(RNNs), and gradient boosting machines, machine learning with a simple algorithm	[42] [85]	Risk stratification was more accurate, and early detection helped with timely interventions. Low costs to perform interventions.	Discrepancies in data and variabilities in the population were challenging factors.
AI for Mortality Prediction	The use of AI in predicting mortality rates in patients with heart failure events is based on the assessment of their data collected from records, tests, and history. Physiological telemetry serves as a low-cost predictor of complications.	Support vector machines, gradient boosting machines, neural networks, multi-sensor noninvasive remote monitoring	[62]	A 93% accuracy in prediction with ECG was seen at Mayo Clinic. HF exacerbation with 76% to 88% sensitivity and 85% specificity.	Intersystem inconsistencies and discrepancies in data were the main challenges faced. Mortality must be evaluated with longer studies.
Medication Management	AI was used in adjusting the dosage, monitoring drug adverse effects, and drug-drug interactions. Personalized pharmacological prescriptions according to the patient.	Machine learning, reinforcement learning	[57]	More precise dosing decreases the number of adverse effects.	Data integration and physician acceptance were the main hurdles. Advances in therapies require constant data entry.
Precision Medicine	Use of AI in reading and analyzing discrepancies in genetics and environmental data to formulate treatment plans accordingly for HF patients. The combination of AI and telemedicine promotes precise care.	Genomic algorithms and deep learning	[97]	Improvement was observed in the stratification of patients based on these factors, enabling a more personalized approach.	The process requires more data at a personal level, which raises ethical concerns, and integration presents a challenge.

**Table 3:** Comparative table of AI methods used in heart failure diagnosis, management, and prognosis.

**AI Approach**	**Description**	**Application in Heart Failure Diagnosis & Prognosis**	**Strengths**	**Limitations**	**References**
Supervised Learning	Models trained on labelled data to predict outcomes.	Applied for ECG signal classification, echocardiography image analysis, and heart failure risk prediction.	High accuracy, explainable models (*e.g.*, Decision Trees, Random Forests).	Large labelled datasets are required; there is a risk of overfitting.	[103, 104]
Unsupervised Learning	Models learn patterns without labelled data.	Used for grouping patients according to risk factors and symptoms.	Effective for discovering hidden patterns; applied in personalized medicine.	It is less interpretable and needs careful validation.	[105]
Convolutional Neural Networks (CNNs)	Deep learning model fine-tuned for image recognition.	Used in echocardiogram image analysis to detect structural abnormalities.	High accuracy in image analysis; automated feature extraction.	Computationally expensive; needs large datasets.	[106]
Recurrent Neural Networks (RNNs) & Long Short-Term Memory (LSTM)	Models designed to analyze time-series data.	Utilized for ECG signal analysis and early detection of heart failure.	Effective for sequential data; captures long-term dependencies.	Vanishing gradient problems; requires high computational power.	[107]
Independent Component Analysis (ICA) & Empirical Mode Decomposition (EMD) + AI	Signal processing techniques combined with AI for feature extraction.	Applied in ECG noise removal and heart failure classification.	Improves signal clarity and classification accuracy.	Computationally intensive; domain expertise required.	[108]

**Table 4:** Comparative performance of AI models in heart failure diagnosis.

**Model**	**Accuracy**	**Recall**	**F1-score**	**Application**
CNN	92.5%	91.8%	92.1%	Echocardiogram image classification [103].
LSTM	89.4%	88.7%	89.0%	ECG signal-based prediction [104].
Random Forest	85.3%	84.1%	84.7%	Risk factor-based heart failure prediction [105].
ICA + AI	94.2%	93.6%	93.8%	ECG noise filtering and classification [106].

## LIST OF ABBREVIATIONS

AI-CDSS: Artificial Intelligence–Clinical Decision Support System
ANN: Artificial Neural Network
CNN: Convolutional Neural Network
CPX: Cardiopulmonary Exercise Test
CRT: Cardiac Resynchronization Therapy
DLA-MSM: Deep Learning Approach with Multi-head Self-attention Mechanism
DLM: Deep Learning Model
ESE: Exercise Stress Echocardiography
GDF-15: Growth Differentiation Factor 15
HFpEF: Heart Failure with Preserved Ejection Fraction
HFrEF: Heart Failure with Reduced Ejection Fraction
HFSS: Heart Failure Survival Score
IoT: Internet of Things
LVEF: Left Ventricular Ejection Fraction
LVSD: Left Ventricular Systolic Dysfunction
MHI: Modified Haller Index
MMP: Matrix Metalloproteinase
MR-proADM: Mid-Regional pro-Adrenomedullin
MR-proANP: Mid-Regional pro-Atrial Natriuretic Peptide
NLP: Natural Language Processing
NNM: Neural Network Model
NT-proBNP: N-terminal pro B-type Natriuretic Peptide
PIIINP: Procollagen Type III N-Terminal Propeptide
PINP: Propeptide of NH2-terminal in Procollagen Type I
RWE: Real World Evidence
SVM: Support Vector Machine
TIMP: Tissue Inhibitor of Metalloproteinases
WGCNA: Weighted Gene Co-expression Network Analysis
